# Designing faculty development to support the evaluation of resident competency in the intrinsic CanMEDS roles: practical outcomes of an assessment of program director needs

**DOI:** 10.1186/s12909-015-0375-5

**Published:** 2015-06-05

**Authors:** Derek Puddester, Colla J. MacDonald, Debbie Clements, Jane Gaffney, Lorne Wiesenfeld

**Affiliations:** 1 Faculty of Medicine, University of Ottawa, 451 Smyth Road, Ottawa, ON K1H 8M5 Canada; 2 Faculty of Education, University of Ottawa, 145 Jean Jacques Lussier, Ottawa, ON K1N 6N5 Canada; 3 Research Consultancy, Ottawa, Canada; 4 Jönköping University, Jönköping, Sweden

**Keywords:** Medical education, CanMEDS, Intrinsic roles, Resident evaluation, Faculty development

## Abstract

**Background:**

The Royal College of Physicians and Surgeons of Canada and the College of Family Physicians of Canada mandate that faculty members demonstrate they are evaluating residents on all CanMEDS (Canadian Medical Education Directions for Specialists) roles as part of the accreditation process. Postgraduate Medical Education at the University of Ottawa initiated a 5-year project to develop and implement a comprehensive system to assess the full spectrum of CanMEDS roles. This paper presents the findings from a needs assessment with Program Directors, in order to determine how postgraduate medical faculty can be motivated and supported to evaluate residents on the intrinsic CanMEDS roles.

**Methods:**

Semi-structured individual interviews were conducted with 60 Postgraduate Program Directors in the Faculty of Medicine. Transcribed interviews were analyzed using qualitative analysis. Once the researchers were satisfied the identified themes reflected the views of the participants, the data was assigned to categories to provide rich, detailed, and comprehensive information that would indicate what faculty need in order to effectively evaluate their residents on the intrinsic roles.

**Results:**

Findings indicated faculty members need faculty development and shared point of care resources to support them with how to not only evaluate, but also teach, the intrinsic roles. Program Directors expressed the need to collaborate and share resources across departments and national specialty programs. Based on our findings, we designed and delivered workshops with companion eBooks to teach and evaluate residents at the point of care (Developing the Professional, Health Advocate and Scholar).

**Conclusions:**

Identifying stakeholder needs is essential for designing effective faculty development. By sharing resources, faculties can prevent ‘reinventing the wheel’ and collaborate to meet the Colleges’ accreditation requirements more efficiently.

## Background

CanMEDS identifies and defines the seven roles required of a competent physician: Medical Expert, Communicator, Collaborator, Manager, Health Advocate, Scholar and Professional. These roles have also been adopted by the College of Family Physicians of Canada (CFPC) to align with their educational programs and accreditation standards [1].

While the roles of Communicator, Collaborator, Manager, Health Advocate, Scholar and Professional are often referred to as the non-medical expert roles by clinicians and educators, the Royal College of Physicians and Surgeons of Canada (RCPSC) have identified these roles as *intrinsic* to the development and a practice of a physician, and advocates that these roles should be called as such [2].

The RCPSC outline and describe appropriate evaluation tools for the CanMEDS roles in *The CanMEDS Assessment Tools Handbook* [3]. Each tool has its own strengths and limitations, and differs in reliability, validity, feasibility and cost [3]. The RCPSC lists individual tools for each CanMEDS role [3], but in practice, they recommend the evaluation of multiple competencies concurrently. This is evident in the recent literature, where there appears to be a concerted effort to incorporate multiple roles into each evaluation (Table 1).

**Table 1:** Examples of assessment tools incorporating multiple CanMEDS roles

Assessment tool	Competencies assessed^a^	Specialty
Encounter Cards/Logs	All roles	General Practice [34]; Emergency Medicine [35]
	All roles except S	Surgery [36]
Field Notes	COM, M, ME	Family Medicine [37]
	All roles	Obstetrics [38]
Multisource Feedback	All roles except HA	Internal Medicine [39]
Objective Structured Clinical Exam	All roles	Neonatal-perinatal [40]; Surgery [41]
	COL, COM, HA, P	Pediatric Hematology & Oncology [42]
Objective Structured Performance-Related Exam	COL, COM, M, ME, P	Surgery [43]
Portfolios	All roles	Diagnostic Radiology [44]; Pediatrics [45]
Simulations	COL, COM, ME	Internal Medicine [46]
Structured Oral Exams	All roles	Neonatal-perinatal [47]
	COM, HA, S	Internal Medicine [48]

^a^*COL* Collaborator, *COM* Communicator, *HA* Health Advocate, *M* Manager, *ME* Medical Expert, *P* Professional, *S* Scholar

When queried about their level of satisfaction with the evaluation of the CanMEDS roles, Program Directors (PDs) ranked the evaluation of the Medical Expert role between satisfied and very satisfied; Communicator, Collaborator, Scholar and Professional between neutral and satisfied; and Manager and Health Advocate ranked between dissatisfied and neutral [4]. This poor ranking for Health Advocate is in alignment with the work of others who suggest this role as being one of the most difficult to teach and evaluate [5, 6]. However both summative and formative evaluation for the Health Advocate role has been proposed utilizing 360° reviews, log books, portfolios and formal evaluations [7].

Evaluation of the Manager role also meets with dissatisfaction from residents [8, 9]. Psychiatry residents suggested several ways to improve current evaluation methods including self-evaluation, reflection papers, quality improvement projects and interdisciplinary team evaluations [8]. Only 5 % of surgical residents thought management topics were well addressed in their programs, but improved their skills after 1-day management seminar [9].

Faculty feedback is a common method of evaluation for the Professional role and higher satisfaction with evaluation was achieved when more people contributed to resident evaluation [10]. Records of unprofessional behavior, incident reports, and patient comments can be used as feedback sources and it is important to triangulate available tools, to reliably and validly evaluate professionalism [11–13].

The Scholar role encompasses diverse competencies, including critical appraisal, research and teaching [3]. For evaluation of clinical teaching skills, several validated instruments have been developed [14, 15]. In-Training Evaluation Reports (ITERs) can evaluate many of the key competencies of this role [3] but may not correlate well with residents’ abilities to publish research [16].

There is limited formal teaching of the Collaborator role, most learning takes place through role modeling, and current methods to evaluate residents have been described as suboptimal [17]. A Collaborator assessment rubric has been developed that may be used for both summative and formative evaluation [18].

The Communicator role can be evaluated reliably and in a structured manner, however new tools continue to be developed that add to the already established literature in the field [3, 19, 20].

In Canadian medical schools, the greatest number of instruments is employed to evaluate the Medical Expert role and PDs report challenges with evaluating the intrinsic roles and find some more difficult to evaluate than others [4]. However, the RCPSC and the CFPC mandate that faculty members demonstrate they are evaluating residents on *all* CanMEDS roles as part of the accreditation process.

The Postgraduate Medical Education (PGME) Office at the Faculty of Medicine at the University of Ottawa (uOttawa) initiated a 5-year quality improvement project to develop and implement a comprehensive system to help faculty members at uOttawa conduct thorough, multi-source evaluations of residents on the intrinsic CanMEDS roles. It was felt that a quality improvement project was required to (a) ensure fair, consistent, transparent evaluations of residents from multiple sources; and, (b) attain and retain accreditation from the Royal College of Physicians and Surgeons of Canada or the College of Family Physicians of Canada.

Phase one of the project involved conducting a needs assessment with the Directors of the PGME programs to understand what faculty members need in order to meet the colleges’ expectations with regard to evaluating residents on the intrinsic roles. The outcomes of this phase included identifying:

1. Enablers and challenges of evaluating the intrinsic roles
2. The Colleges’ expectations for evaluating the intrinsic roles
3. Strategies and resources that can be used to support faculty when evaluating residents
4. Best practices for evaluating resident competency related to the intrinsic roles.

This paper presents the findings from the needs assessment with PDs, in order to determine how postgraduate medical faculty could be motivated and supported to evaluate resident competency in each of the intrinsic roles. Conducting a needs analysis and addressing stakeholders’ concerns when making program decisions is an essential step in designing and delivering quality faculty development and getting buy in from stakeholders when implementing the program [21, 22].

## Methods

This qualitative study used purposeful sampling by inviting all 68 PDs in the Faculty of Medicine at uOttawa to take part in the interviews. This convenient sampling provided a representative sample to identify the needs of faculty members in all disciplines in the Faculty. There was no bias in selection of participants.

All 68 PDs were emailed and invited to participate in a telephone interview at their convenience. A second email was sent two weeks later to those who did not respond to the first email. Sixty of the 68 PDs were available for, and agreed to participate in, an interview. Eight PDs did not respond to either of the two email invitations.

The purpose of the interviews was to (a) determine current reported practices with evaluating the intrinsic roles, (b) identify enablers and challenges to evaluating these roles, and (c) identify how the central PGME office can support programs with evaluating the intrinsic roles and meet expectations for the RCPSC and the CFPC accreditation criteria.

The 60 interviews were conducted between February 28th and June 14th 2013 by the Special Project Lead, Innovation and Evaluation, PGME. The first author of this paper conducted all 60 interviews. The interviewer is a clinical psychiatrist and researcher with courses in qualitative interviewing methods (Masters in Education). The interviewer was a colleague of the faculty members and held no power over them. No sensitive issues were addressed during the interviews and it was felt that with 60 participants one data source would suffice to represent the needs of the faculty in order to develop practical means to support faculty in evaluating residents on the intrinsic roles. The same semi-structured questionnaire (Appendix A) was used in each interview to ensure consistency between, and among, the 60 interviews.

All interviews were audio recorded with the permission of the interviewees and transcribed verbatim. The interviews lasted from 5 to 25 min. The average interview was 14 min. Interview data was stored on a password-protected computer and only accessible by researchers in this study.

Qualitative data analysis was conducted by the second author in this study, and guided by established methodology [23, 24]. Member checks were completed with the principal investigator who conducted the interviews. The principal investigator checked the data analysis by reflecting on the interviews and verifying them with the interview transcripts.

The interview transcripts were checked for accuracy by the researcher listening to the audio recording (mp3 file) and comparing them to the transcribed text. Open coding of the text was then performed by hand. After a preliminary list of codes was developed, the transcripts were coded a second time to group common codes together to form themes. The coding was reviewed several more times to ensure that no new codes emerged from the data. Once the themes reflected “the recurring regularities or patterns in the study” ([23] p181), and the researcher was satisfied the themes reflected the views of the participants, the data were assigned to categories to provide rich, detailed, and comprehensive information that would indicate what faculty need in order to effectively assess their residents on the intrinsic roles.

Relevant information from the emerging themes was used to weave a story from the 60 PDs’ perspectives portraying current practices, enablers and challenges, and how the PGME office can support departments with evaluating the intrinsic roles and meet the expectations for the RCPSC and the CFPC accreditation criteria. Direct quotations are used throughout this paper to allow participants’ voices to be heard and to obtain objective evidence regarding the participants’ perceptions of evaluating the intrinsic roles.

All interview participants were provided a copy of the qualitative data analyses to ensure the interpretation was according to their intentions and perspective, and to provide permission to use their data in reporting. Interview participants were provided an opportunity to adapt, remove or elaborate on any quote or text that misrepresented their perspective. Participants were notified of the intent to publish, and were asked to let the authors know if they had issues with publication. Participants were informed that by not responding they were agreeing to the manuscript being published. Due to the fact that ethics approval was not needed for this quality improvement project, no power situation occurred (faculty interviewed faculty); no sensitive information was gathered and to accommodate the busy schedules of clinical physicians, this was deemed to be a fair indication of their approval. A few participants made minor edits to the report and no one identified issues with the report being published.

### Ethics statement

The research proposal was reviewed by the OHSN-REB (Ottawa Health Science Network Research Ethics Board) and it was determined our research was a program evaluation and therefore ethics approval was not required.

## Results

All 60 PDs interviewed welcomed a coordinated effort from the PGME office to support them in evaluating the CanMEDS roles. Several PDs expressed appreciation for the initiative to cross-pollinate ideas and develop consistency and standards across the 68 departments at the Faculty of Medicine. The findings from the interviews are organized under three broad themes: State of Affairs, Challenges, and Needs. Each theme has several sub-themes, discussed in the ensuing sections.

### State of affairs

Four sub-themes emerged with regard to the state of affairs: Current strategies, Accreditation driven, Superficial, and Health advocacy.

#### Current strategies

Repeatedly, PDs admitted that the process for evaluating the intrinsic competencies was informal, inconsistent, subjective, and lacked structure. While the 60 PDs provided a list of strategies they currently use to evaluate residents (encounter cards, field notes, multisource feedback, ITERs, one45, resident portfolios, simulation mannequins and journal clubs), the most common response provided was ‘informal observation’.

#### Accreditation driven

PDs were unabashed about the fact that their motivation for improving faculty’s effectiveness at evaluating the CanMEDS roles was accreditation driven. One PD voiced: “Word is out … evaluating CanMEDS roles is going to be very important as to whether or not somebody gets accredited”. Similarly, a PD acknowledged he feared the lack of documentation may be an issue in their upcoming accreditation:

The College comes to accredit our program and … if they ask how I evaluated professionalism I won’t have anything to show. It is not documented anywhere. … I don’t know how we are going to fix it because we are all swamped.

#### Superficial

PDs reported evaluation of the intrinsic roles was ‘superficial’. One PD explained: “I have very good trainees. When I get their ITER forms … everything is skewed … everything is outstanding. They cannot all be outstanding”. Another director suggested that often the evaluation process is quite subjective: “We watch them talk to patients and if the patients are reasonably satisfied then they are rated as ‘good communicators’. We don’t have any measuring sticks”. One PD suggested faculty members may evaluate residents highly to avoid controversy. “There is a lot of fear as to what reprisals can happen both for the trainees as well as for yourself”.

#### Health advocacy

PDs reported their faculty members need help with all the intrinsic roles but health advocate emerged as being the most difficult to teach and evaluate. One director disclosed: “I feel like assessing or teaching advocacy is always challenging …. other than to say that they have done an advocacy project and check a checkbox”. Similarly, a PD stated he didn’t know how to appropriately evaluate health advocacy. “That piece I don’t think we are doing well.”

### Challenges

PDs identified four challenges to enabling their faculty to effectively evaluate the CanMEDS roles: Prerequisite needs, Time, Perceived value, and Incentive.

#### Prerequisite needs

Several PDs suggested there were needs that had to be addressed before they could expect their faculty members to be able to evaluate the intrinsic roles. One PD stated:

“…the requisite step to being able to evaluate the intrinsic roles is being aware of what they are and ensuring they are part of the curriculum”.

When asked how the Faculty of Medicine can help support its members, one director stated: “Some leadership and guidelines”. PDs pointed out they need clear benchmarks for where a resident is expected to be at various stages of their training. Another PD identified not knowing how to teach as a ‘gap’. “The fact that they don’t know how to teach the roles becomes a barrier to properly evaluating it”. Finally a PD pointed out that teaching the intrinsic roles had to move beyond role modeling in order to meet accreditation expectations.

#### Time

When asked about barriers to evaluating residents on the intrinsic roles, one PD stated, “Lack of time”. Some of the specialty PDs expressed frustration with finding the time to spend on the intrinsic competencies. “Many of us are concerned that the reduction in the attention towards the Medical Expert competency is depriving us of the ability to actually train competent [specialists]”. Another PD stated: “We don’t sit around the table trying to figure out how we put a program together to evaluate health advocacy. Maybe we should. But … there are only so many hours in a day”. Finally, PDs reported the faculty members sometimes feel they spend insufficient time with the resident: “…many staff feel they don’t get a chance to work with the residents for that long to be able to say what’s going on and how they are doing with everything”.

#### Perceived value

Approximately fifty of the sixty PDs reported the Faculty of Medicine culture and residents tend to place higher value on the Medical Expert role. One PD stated: “The eyes gloss over a little bit and it may not be as meaningful to the resident as when they receive their Medical Expert feedback”.

PDs were unapologetic for focusing their evaluation on the Medical Expert role as they noted this is the role the RCPSC exam focuses on. A few PDs insinuated there were generational issues: “…we have physicians who have been around 20–30 years. They are hard to get feedback from outside of the Medical Expert role … it is just not something that they trained with and that they buy into”. Another PD discussed the difficulty of evaluating senior residents: “Once they reach the R-6 and R-7 year they are tired of being evaluated and want to be working. They are not going to the sessions on the non-medical roles we are scheduling”.

#### Incentive

PDs identified lack of incentives as a challenge for faculty to evaluate the intrinsic roles. When asked what they need to support their faculty members, one PD revealed:

As PD [the only thing] I have is to basically use a stick to say ‘fill in your evaluations or I’m going to nag you to death’. It would be nice if I could recognize faculty doing a good job and provide them with compensation whether financially driven, promotional based or a career advantage.

Another PD agreed that compensation was needed as motivation for faculty members. “Financial incentives always work”.

### Needs

PD needs with regard to evaluating residents on the CanMEDS roles emerged into six themes: Support, Faculty-wide approach, Tools, Technology and point of care, Faculty development, and Standardized.

#### Support

When asked what PDs need to support faculty members in becoming more effective at evaluating the CanMEDS roles, resources in the form of time and money repeatedly emerged. One PD asked for, “Suggestions on how to document. We just don’t know how to write it down or tick it off”. PDs reported faculty members want evaluation made easy by having ‘prepared templates’ on the intrinsic competencies.

#### Faculty-wide approach

PDs want courses and evaluation strategies developed and delivered across the Faculty so they are not ‘reinventing the wheel’. PDs stated strategies do not need to be program specific and collaboration across different departments would be an intelligent use of resources. One PD communicated: “We are all facing the same challenges. I think the PGME office is the one unifying force”. PDs welcomed procedures and strategies among national specialty departments. One director professed: “Whatever we do in Calgary we should be doing in Ottawa. The residents are all writing the same exams so why don’t we have all the same evaluations”.

#### Tools

One of the biggest challenges PDs identified was a lack of available evaluation tools:

“We are still searching for assessment tools for these roles. Tools that can be easily and readily applied”. PDs said they were left to their own devices when it came to trying to “create, beg, borrow and steal” tools. PDs suggested they need a ‘toolbox’ of useful easy-to-use instruments to evaluate each of the CanMEDS roles.

#### Technology and point of care

PDs were clear that the evaluation tools need to be simple to use and ‘available at the point of care’ on mobile devices. Other PDs suggested sharing resources and evaluation tools on a website or a repository. PDs emphasized that it is a challenge when FD is offered at inconvenient times. A few PDs mentioned web conferencing. Another PD suggested delivering the information in a flexible format “… get people to do things where they may not want to travel and give up clinic time”.

#### Faculty development

PDs consistently revealed they need more faculty development (FD) on what the intrinsic roles are, and how to effectively teach and evaluate them. Training should be ‘hands-on’, ‘case-based’ with ‘real world examples’. PDs suggested faculty should be held to the same standard as residents with regard to keeping up to date: “The teaching world is changing. So it makes sense that people need training. … These are not only core competencies for our learners but also for our teachers”.

However, some PDs were cognizant of the fact that it is often hard to get ‘bums in seats’ even when FD is offered. One PD suggested: “Things that … we can discuss quickly in rounds may be more useful than larger groups events”. PDs stated they would like ‘convenient in-house’ FD.

#### Standardized

PDs suggested the importance of developing standardized formal evaluation tools to reduce bias and increase quality standards. One PD pointed out that when the evaluation of the intrinsic roles shifted from informal observation to a formal evaluation, issues with the resident’s competency arose for the first time:

One of my trainees had to do a formal history and physical assessment. Her evaluations prior were much more observational and always ‘above expectations’. When she came to a formal assessment of those skills this trainee had significant issues. I wonder with all the other intrinsic roles if we go deeper down are those skills actually there.

PDs noted that formal evaluation including documentation is essential especially when something goes wrong:

You hear things along the way but no one actually documents it. And then it suddenly blows up. But all along there were hints but it is either not documented or it is not tracked sufficiently and you run into a situation.

PDs stated having similar evaluation strategies across departments would be a first step toward standardizing how the intrinsic roles are being evaluated.

## Discussion

The findings from the interviews revealed PDs appreciated the initiative to develop consistency and standards across the departments at the Faculty of Medicine with regard to evaluating residents on the intrinsic roles. PDs claimed their departments were effective at evaluating the Medical Expert role, but were not doing an adequate job at evaluating the intrinsic roles. PDs consistently cited Medical Expert as the role the Colleges’ exam focuses on, and the role that demands the most time to master. Other authors have found similar results, with PDs challenged with assessing the intrinsic roles [4].

The original purpose of the project was to conduct a needs analysis with PDs to identify what their faculty members need in order to evaluate residents on the intrinsic roles. However, in the needs analysis it was discovered that some faculty members needed information on what the intrinsic roles are, and most faculty need strategies and resources on how to teach the intrinsic roles, before they can be expected to evaluate residents on them.

Several PDs acknowledged their departments struggle with taking time away from medical procedures to teach and evaluate the intrinsic roles. Faculty overload has been recognized as a barrier to implementation of CanMEDS [25]. Time is also listed as a reason for why residents don’t engage in health advocacy [5, 26]. If residents and PDs both indicate lack of time as a limitation to intrinsic competencies, this is a significant barrier that must be addressed to successfully implement teaching and evaluation of the intrinsic roles.

Although there are existing tools available to evaluate residents on the intrinsic roles ([3], Table 1), many of these tools are discipline-specific and would need to be adapted to be used in another department. Faculty reported being too busy to look for, or adapt, existing tools. In addition, it has been reported that faculty members often lack the confidence to produce appropriate evaluation tools [27]. Moreover several faculty members said they were not aware of available assessment tools.

Standardization of tools and objectivity is critical according to the PDs, even though many listed current evaluation strategies as being informal, inconsistent, subjective, and lacking structure. Competency-based medical education demands continuous and frequent evaluation; methods that are criterion-based; assessment tools that meet minimum standards of quality; and assessment that utilizes multi-source feedback and involves active engagement by the trainee [28]. When designing and developing an overall evaluation plan, reproducibility, equivalence, feasibility, educational effect, catalytic effect and stakeholder acceptance must be respected [29].

Many PDs cited the need to not “reinvent the wheel” and proposed a faculty-wide approach, using evaluation tools that were not necessarily program specific but that could be tailored to their own specialties. In addition, PDs were clear that evaluation tools should be simple to use and available at the point of care. Online tools and those on mobile devices were suggested to keep pace with the expectations of this generation of residents.

PDs welcomed strategies among national specialty departments or departments within the faculty to establish benchmarks, matrixes and instruments to effectively evaluate the CanMEDS roles. PDs from Canadian, English-speaking medical schools have expressed a similar desire for national collaboration on resident evaluation between specialty programs [30]. PDs suggested this approach would be a first step to standardizing evaluation, and may consequently, strengthen the entire Faculty of Medicine.

Identifying faculty needs was the initial step in this multi-year project. The next step involves supporting faculty to teach and evaluate the intrinsic roles at all the Royal College’s competence-by-design residency stages of training. It became clear from the findings in the needs analysis that faculty development would be most effective if it included case studies with practical ideas on how to integrate teaching the intrinsic roles into teaching the Medical Expert roles, as well as providing evaluation tools conveniently available at the point of care on a mobile device.

These findings informed us to provide convenient FD with companion eBooks full of teaching and evaluation resources that can be accessed on mobile devices at the point of care. The first three workshops and eBooks ([31–33]; http://ipad-fm.ca/pgmeebooks) were presented at the annual FD day on May 14, 2014. We hope by making these resources available, other faculty can benefit from our efforts and best practice, and help others from ‘reinventing the wheel’.

Our study was complicated by the fact that while we were trying to address the needs of faculty in order to support them evaluating residents on the intrinsic roles, the RCPSC was revising the CanMEDS roles. Our research team remained in contract with the RCPSC and our faculty development solutions align with the latest version of the CanMEDS 2015 initiatives. For example, the workshop and eBook currently under construction will be entitled ‘Leader’ instead of ‘Manager’ to coincide with the newest version of the CanMEDS framework. Other universities and institutions are showing interest in this study. The eBooks have been utilized to train physicians in Oman and Saudi Arabia and the Royal College of Physicians and Surgeons of Canada have shown interest in their use.

The next steps in this project include re-offering the workshops with their companion eBooks at convenient department workshops to increase the number of participating faculty. In addition, the remaining three workshops and eBooks are under construction (Leader, Communicator and Collaborator). Finally, all workshops with their companion eBooks will be evaluated by participating faculty to determine if they improve the prevalence and effectiveness of faculty evaluating and documenting residents’ competencies with regard to the intrinsic roles.

## Conclusions

In order to support and motivate postgraduate medical faculty to evaluate residents on the intrinsic CanMEDS roles, it is essential to:

- Provide faculty development that defines the intrinsic roles and supply practical ideas and resources on how to teach the roles, before expecting faculty to be able to evaluate them.
- Demonstrate how the intrinsic roles can be integrated into teaching the Medical Expert role in response to time constraints of faculty members.
- Provide practical resources, at the point of care, to make teaching and evaluating the intrinsic roles as convenient and authentic as possible.

## Appendix A

Interview Questions for Program Directors

1. How do your faculty members currently assess their resident’s competencies with regard to the following CanMEDS roles (collaborator; communicator; scholar; manager; health advocate and professional)?
2. What tools or instruments do your faculty currently use to assess their resident’s competencies with regard to the CanMEDS roles?
3. What barriers and/or challenges have your faculty members identified or experienced with regards to using assessment tools in the teaching and learning environment?
4. What gaps have your faculty members identified in assessing residents competencies with regard to the CanMEDS roles?
5. Have your faculty members identified or adhered to any best practices with regard to assessing the CanMEDS roles?
6. What do your faculty members need to be more effective at assessing their resident’s competencies with regard to the CanMEDS roles?
7. How can we support your faculty to be more effective at assessing their resident’s competencies with regard to the CanMEDS roles?

## Abbreviations

CanMEDS: Canadian Medical Education Directions for Specialists
CFPC: College of Family Physicians of Canada
FD: Faculty development
ITER: In-training evaluation report
OHSN-REB: Ottawa Health Science Network Research Ethics Board
PD(s): Program director(s)
PGME: Postgraduate medical education
RCPSC: Royal College of Physicians and Surgeons of Canada
uOttawa: University of Ottawa
